# Environments affect blood pressure in toddlers: The Japan Environment and Children’s Study

**DOI:** 10.1038/s41390-023-02796-8

**Published:** 2023-08-26

**Authors:** Keita Kanamori, Tomohisa Suzuki, Nozomi Tatsuta, Chiharu Ota, The Japan Environment and Children’s Study Group, Michihiro Kamijima, Shin Yamazaki, Yukihiro Ohya, Reiko Kishi, Nobuo Yaegashi, Koichi Hashimoto, Chisato Mori, Shuichi Ito, Zentaro Yamagata, Hidekuni Inadera, Takeo Nakayama, Tomotaka Sobue, Masayuki Shima, Seiji Kageyama, Narufumi Suganuma, Shoichi Ohga, Takahiko Katoh

**Affiliations:** 1 Department of Development and Environmental Medicine, Tohoku University Graduate School of Medicine, Sendai, Japan; 2 Department of Pediatrics, Iwate Prefectural Iwai Hospital, Ichinoseki, Japan; 3 Department of Pediatrics, Tohoku University Hospital, Sendai, Japan; 4 Environment and Genome Research Center, Tohoku University Graduate School of Medicine, Sendai, Japan; 5 Nagoya City University, Nagoya, Japan; 6 National Institute for Environmental Studies, Tsukuba, Japan; 7 National Center for Child Health and Development, Tokyo, Japan; 8 Hokkaido University, Sapporo, Japan; 9 Tohoku University, Sendai, Japan; 10 Fukushima Medical University, Fukushima, Japan; 11 Chiba University, Chiba, Japan; 12 Yokohama City University, Yokohama, Japan; 13 University of Yamanashi, Chuo, Japan; 14 University of Toyama, Toyama, Japan; 15 Kyoto University, Kyoto, Japan; 16 Osaka University, Suita, Japan; 17 Hyogo Medical University, Nishinomiya, Japan; 18 Tottori University, Yonago, Japan; 19 Kochi University, Nankoku, Japan; 20 Kyushu University, Fukuoka, Japan; 21 Kumamoto University, Kumamoto, Japan

## Abstract

**Background:**

The primary objective of this study was to examine risk factors for toddler’s hypertension.

**Methods:**

Subjects of this study were children and parents participating in a national birth cohort study in Japan, the Japan Environment and Children’s Study. We measured the children’s blood pressure (BP) at 2 and 4 years old. We obtained children’s and parents’ backgrounds from the questionnaire. We investigated the factors that affect BP elevation.

**Results:**

Within 4988 participants, the mean systolic BP at 2 years old was 91.2 mmHg for boys and 90.0 mmHg for girls. The mean systolic BP at 4 years old was 93.8 mmHg for boys and 93.1 mmHg for girls. Parental smoking was associated with elevated values of BP at 2 and 4 years old. Obesity, gestational hypertension, and parental lower education were associated with elevated values of BP at 4 years old. Hypertensive group had a significantly higher obesity rate. The mother’s lower education and parental smoking were involved in hypertensive groups.

**Conclusion:**

Parental smoking had a significant effect on BP even in early toddlers. We emphasize the importance of avoiding second-hand smoking from early infancy to prevent future lifestyle-related illnesses including hypertension.

**Impact:**

- The mean systolic BP at 2 years old was 91.2 mmHg for boys and 90.0 mmHg for girls.
- The mean systolic BP at 4 years old was 93.8 mmHg for boys and 93.1 mmHg for girls.
- Obesity, parental smoking, and lower education were associated with hypertension at 4 years old.
- Parental smoking was associated with hypertension at 2 and 4 years old.
- We emphasize the importance of avoiding second-hand smoking from early infancy.

## Introduction

Hypertension in childhood is a risk factor for hypertension and cardiovascular disease in adulthood.^1–3^ To screen pediatric blood pressure (BP), it is necessary to clarify normal BP in childhood. Flynn et al. reported normal BP in preschool children in the United States.^4^ On the other hand, in Japan, since BP measurement is not included in general infant/toddler health examinations, the reports were limited to school children.^5–8^ Considering that BP is variable in the races and lifestyles,^9^ it is important to estimate normal BP value in the infant/toddler population.

In adults, lifestyle factors such as a high-salt diet, lack of exercise, obesity, alcohol, and smoking were known risk factors for hypertension.^10–15^ Some studies have shown a correlation between obesity and hypertension in children,^16–20^ and Simonetti et al. also reported that several factors such as obesity and parental smoking were at risk for hypertension of preschool children aged 5–6 years.^21^ In addition, some reports showed that environmental pollutants such as ochratoxin A,^22^ mercury, cadmium^23^ and mono-benzyl phthalate^24^ played an important role in the etiology of hypertension of adolescents besides obesity. However, there were few reports examining hypertension and risk factors in toddlers, 4 years or less.

In Japan, a nationwide large birth cohort study to clarify the relationship between environmental change and child health, the Japan Environment and Children’s Study (JECS), was conducted between January 2011 and March 2014. We aimed to examine the risk factors for hypertension in toddlers with BP data collected in JECS.

## Methods

### Participants and the study protocol

The JECS protocol, which has been published elsewhere,^25^ was reviewed and approved by the Ministry of the Environment’s Institutional Review Board on Epidemiological Studies and by the Ethics Committees of all participating institutions (Ethical Number: 100910001). All the procedures and experiments were performed after receiving written informed consent from all the participants. Among all the participants, a sub-cohort study including face-to-face assessment of neuropsychiatric development, body measurement, pediatrician’s examination, and blood/urine collection for clinical/chemical analysis was conducted in randomly selected children aged 2 and 4 years old.^26^ We measured the children’s BP, height and weight at 2 and 4 years of age. After resting children for about 5 min, a nurse or doctor measured BP three times and adopted the median value, but for children who could not participate in the three measurements, we adopted the median value among the measured values. We used Baxter’s Welch Allyn® Gold Series DS66 Trigger Aneroids. The cuff used in this study has a width equal to or greater than 40% of the circumference of the upper arm. In addition, the cuff has a length that encloses 80% or more of the upper arm circumference. The ratio between the width and length of the cuff is 1:2 or greater. In cases who were crying or moving when measuring BP, the measured values were excluded. We defined the hypertensive group as children with systolic BP (SBP) ≥95th percentile, following previous research on hypertension in children.^18,19^ We calculated the standard body weight by using the following formula based on the infant physical growth survey of the Ministry of Health, Labour Standards in Japan; boys: 0.00206 × weight (kg)^2^ – 0.1166 × weight (kg) + 6.5273, girls: 0.00249 × weight (kg)^2^ – 0.1858 × weight (kg) + 9.0360.^27^ We calculated body surface area (BSA) from height and pre-pregnancy weight by using the Du Bois formula.^28^ We obtained child’s birth weight, mother’s height and weight, father’s height and weight, child’s past history, gestational hypertension (GH), mother’s smoking, father’s smoking, mother’s alcohol consumption, mother’s educational background, and father’s educational background from the questionnaire at the age of 3 years, and serum thyroid stimulating hormone (TSH) and free thyroxine (fT4) were measured at the age of 2 years. The exposure characteristics of the JECS participating mothers were summarized elsewhere.^29^

### Statistical analysis

We presented the descriptive statistics value for boys and girls at 2 and 4 years old and investigated the risk factors for hypertension with the datasets, jecs-ta-20190930 and jecs-qa-20210401. The dataset jecs-qa-20210401 had 104,062 records and the dataset jecs-ta-20190930 had 104,059 records. Since the participants’ characteristics differ depending on sex in general (Table 1), we analyzed boys and girls separately. We calculated correlation coefficients between BP and the continuous variables and tested the difference in BP by using Student’s *t*-tests if the explanatory variables were nominal variables. In addition, we performed multiple regression analysis. As explanatory variables in multiple regression analysis, we selected sex, parental education level and smoking, birth weight and children’s body mass index (BMI), which were shown as classical risk factors for hypertension in many reports,^16–21,30–34^ in addition to the items for which significant differences were observed in this study. We also divided children into three groups in terms of parental smoking status: both nonsmoking, one smoking, or both smoking, and performed a one-way analysis of variance on parental smoking. In addition, we compared backgrounds between hypertensive and non-hypertensive groups by Student’s *t*-tests or Pearson’s *χ*^2^ test.

**Table 1:** Characteristics of participants, *n* = 4988.

	Boys (*n* = 2541)	Girls (*n* = 2445)	*p* value
SBP, 2 yo (mmHg)	91.2 ± 8.44	90.0 ± 8.09	<0.05
DBP, 2 yo (mmHg)	51.6 ± 8.05	51.7 ± 7.97	0.732
SBP, 4 yo (mmHg)	93.8 ± 7.77	93.1 ± 8.10	<0.05
DBP, 4 yo (mmHg)	54.2 ± 7.58	54.6 ± 7.86	0.148
Birth weight (g)	3084 ± 424	2994 ± 398	<0.05
Height, 2 yo (cm)	84.5 ± 2.99	83.3 ± 2.90	<0.05
Weight, 2 yo (kg)	11.7 ± 1.21	11.2 ± 1.17	<0.05
Obesity rate, 2 yo	2.77 ± 7.41	3.49 ± 7.67	<0.05
BSA, 2 yo (m^2^)	0.51 ± 0.03	0.50 ± 0.03	<0.05
BMI, 2 yo	16.4 ± 1.17	16.2 ± 1.18	<0.05
Height, 4 yo (cm)	100.3 ± 3.79	99.3 ± 3.72	<0.05
Weight, 4 yo (kg)	15.6 ± 1.79	15.4 ± 1.78	<0.05
Obesity rate, 4 yo	0.24 ± 7.36	1.05 ± 7.64	<0.05
BSA, 4 yo (m^2^)	0.65 ± 0.05	0.64 ± 0.05	<0.05
BMI, 4 yo	15.5 ± 1.13	15.5 ± 1.18	0.648
TSH (µIU/mL)	2.48 ± 1.27	2.29 ± 1.21	<0.05
fT4 (pg/mL)	1.22 ± 0.14	1.20 ± 0.13	<0.05
Mother’s height (cm)	158.2 ± 5.36	158.5 ± 5.51	0.131
Mother’s weight (kg)	53.2 ± 8.62	53.4 ± 9.07	0.364
Mother’s BMI	21.2 ± 3.23	21.3 ± 3.30	0.860
Father’s height (cm)	171.7 ± 5.81	171.8 ± 5.88	0.419
Father’s weight (kg)	69.1 ± 11.11	69.7 ± 11.14	0.077
Father’s BMI	23.4 ± 3.44	23.6 ± 3.40	0.131
Past history (*n*, %)	122 (4.9)	90 (3.8)	0.058
Gestational hypertension (*n*, %)	28 (1.1)	22 (0.9)	0.566
Mother’s smoking (*n*, %)	79 (3.1)	71 (2.9)	0.726
Mother’s drinking (*n*, %)	281 (11.1)	253 (10.4)	0.439
Mother’s low education (*n*, %)^a^	748 (29.6)	794 (32.6)	<0.05
Father’s smoking (*n*, %)	999 (39.9)	1029 (42.7)	<0.05
Father’s low education (*n*, %)^a^	965 (38.3)	967 (39.8)	0.282

Continuous variables: mean ± standard deviation.

*BMI* body mass index, *BSA* body surface area, *DBP* diastolic blood pressure, *fT4* free thyroxine, *SBP* systolic blood pressure, *TSH* thyroid stimulating hormone.

^a^Highest level of education was high school or below.

We conducted the statistical analysis with R (Version: 3.3.0+). We confirmed the normality of continuous variables by histogram and quantile–quantile plot. The significance level was set to *p* value <0.05.

## Results

The total number of participants was 4988 in this study with 2541 boys, 2445 girls, and 2 unspecified. A total of 212 (4.2%) had past medical histories. When classified according to ICD-11^35^ there were 67 cases of “developmental anomalies,” 62 cases of “diseases of the nervous system,” and 25 cases of “diseases of the immune system.” The remaining categories had less than 10 cases each. Among “developmental anomalies,” “structural developmental anomalies of the circulatory system” were the most frequent, with ventricular septal defect being the most common disease with 16 cases. Out of the 62 cases of “diseases of the nervous system,” 53 were febrile seizure, which was the most common disease. Among the 25 cases of “diseases of the immune system,” 23 were Kawasaki disease, the second most common disease after febrile seizure. Of a total number of 26,012 BP measurements, 22,813 were measured during resting or sleeping, and 3199 were measured during crying or moving. Supplementary Table 1 shows a comparison of BP during resting/sleeping versus during crying/moving. SBP and diastolic BP (DBP) at 2 years old and SBP at 4 years old increased significantly in both boys and girls during crying/moving while DBP at 4 years old decreased significantly. For the following analysis, we excluded BP value measured during crying/moving. Table 1 shows the characteristics of the participants. The mean SBP at 2 years old was 91.2 ± 8.4 mmHg (mean ± standard deviation) for boys and 90.0 ± 8.1 mmHg for girls, respectively, and was significantly higher in boys. The mean SBP at 4 years old was also significantly higher in boys: 93.8 ± 7.8 mmHg and in girls: 93.1 ± 8.1 mmHg, respectively. There was no significant difference in mean DBP between boys and girls in both age groups. For boys, median SBP at 2 years old was 90 mmHg (interquartile range [IQR] 86–97), median DBP at 2 years old was 50 mmHg (IQR 46–58), median SBP at 4 years old was 94 mmHg (IQR 88–99), median DBP at 4 years old was 54 mmHg (IQR 50–60). For girls, median SBP at 2 years old was 90 mmHg (IQR 84–96), median DBP at 2 years old was 50 mmHg (IQR 46–58), median SBP at 4 years old was 92 mmHg (IQR 88–98), median DBP at 4 years old was 56 mmHg (IQR 50–60).

Table 2 shows the correlation coefficient between continuous variables and BP in each age/sex group. There were weak correlations between SBP and weight (*r* = 0.23) and SBP and BSA (*r* = 0.22), respectively, in 4-year-old boys, without any strong correlation for other variables. Tables 3 and 4 show the relationship between nominal variables and BP in each age/sex group. SBP in 2-year-old boys and girls and SBP and DBP in 4-year-old girls were significantly elevated when one or both parents were smokers. SBP in 4-year-old boys and girls and DBP in 4-year-old girls was significantly elevated when the mother’s highest level of education was high school or below, and SBP in 2-year-old girls and 4-year-old boys and girls was significantly elevated when the father’s highest level of education was high school or below. SBP in 4-year-old boys and girls was also significantly elevated when the mothers had GH. The presence or absence of past medical histories did not significantly affect BP. Table 5 shows the results of the multiple regression analysis. BMI was associated with significant differences in both SBP and DBP for all ages. Sex and parental smoking showed significant differences in SBP for all ages. Mother’s low education showed significant differences in 4-year-old SBP and DBP. GH was associated with significant differences in 4-year-old SBP.

**Table 2:** Correlation coefficient with blood pressure.

	2 yo, Boys	2 yo, Girls	4 yo, Boys	4 yo, Girls
SBP				
Birth weight	0.038	0.0094	0.091	0.013
Height	0.086	0.076	0.17	0.10
Weight	0.17	0.13	0.23	0.16
Obesity rate	0.17	0.12	0.19	0.14
BSA	0.15	0.12	0.22	0.15
BMI	0.16	0.11	0.17	0.13
TSH	–0.014	0.0068	–0.0020	0.0062
fT4	–0.017	0.017	0.053	0.0048
Mother’s height	0.040	–0.0060	0.036	0.039
Mother’s weight	0.080	0.064	0.18	0.079
Mother’s BMI	0.067	0.076	0.11	0.067
Father’s height	–0.0088	–0.010	0.042	–0.022
Father’s weight	0.028	0.051	0.12	0.071
Father’s BMI	0.038	0.060	0.11	0.089
DBP				
Birth weight	0.011	0.041	0.055	0.049
Height	0.057	0.086	0.10	0.073
Weight	0.11	0.14	0.14	0.14
Obesity rate	0.10	0.11	0.095	0.14
BSA	0.096	0.12	0.13	0.13
BMI	0.096	0.10	0.083	0.13
TSH	–0.0087	0.0038	–0.0050	–0.014
fT4	–0.011	0.015	0.038	0.0028
Mother’s height	0.019	0.018	0.028	0.047
Mother’s weight	0.015	0.012	0.073	0.043
Mother’s BMI	0.0072	0.0091	0.064	0.025
Father’s height	0.020	0.011	0.024	–0.029
Father’s weight	–0.026	0.029	0.050	0.045
Father’s BMI	–0.033	0.027	0.043	0.060

*BMI* body mass index, *BSA* body surface area, *DBP* diastolic blood pressure, *fT4* free thyroxine, *SBP* systolic blood pressure, *TSH* thyroid stimulating hormone.

**Table 3:** Blood pressure between two groups in boys (mmHg).

	Yes (*n*)	No (*n*)	*p* value
SBP, 2 yo			
Past history	91.1 ± 8.06 (122)	91.2 ± 8.46 (2359)	0.89
Gestational hypertension	90.2 ± 7.34 (28)	91.2 ± 8.45 (2513)	0.55
Parental smoking	91.9 ± 8.50 (1010)	90.8 ± 8.39 (1492)	<0.05
Mother’s drinking	91.6 ± 8.51 (281)	91.2 ± 8.42 (2249)	0.44
Mother’s low education^a^	91.5 ± 8.66 (748)	91.1 ± 8.36 (1779)	0.28
Father’s low education^a^	91.4 ± 8.47 (965)	91.1 ± 8.44 (1554)	0.48
DBP, 2 yo			
Past history	51.6 ± 7.37 (122)	51.6 ± 8.08 (2359)	0.93
Gestational hypertension	51.0 ± 7.83 (28)	51.6 ± 8.05 (2513)	0.73
Parental smoking	51.9 ± 7.95 (1010)	51.4 ± 8.08 (1492)	0.13
Mother’s drinking	51.3 ± 7.66 (281)	51.6 ± 8.09 (2249)	0.58
Mother’s low education^a^	51.4 ± 8.27 (748)	51.6 ± 7.95 (1779)	0.59
Father’s low education^a^	51.6 ± 8.12 (965)	51.6 ± 7.99 (1554)	0.98
SBP, 4 yo			
Past history	94.1 ± 8.09 (122)	93.8 ± 7.77 (2359)	0.64
Gestational hypertension	97.8 ± 7.65 (28)	93.8 ± 7.77 (2513)	<0.05
Parental smoking	94.1 ± 7.54 (1010)	93.6 ± 7.91 (1492)	0.19
Mother’s drinking	94.0 ± 8.01 (281)	93.8 ± 7.75 (2249)	0.69
Mother’s low education^a^	94.7 ± 7.84 (748)	93.4 ± 7.73 (1779)	<0.05
Father’s low education^a^	94.4 ± 8.00 (965)	93.4 ± 7.62 (1554)	<0.05
DBP, 4 yo			
Past history	54.1 ± 7.31 (122)	54.3 ± 7.61 (2359)	0.78
Gestational hypertension	55.3 ± 7.06 (28)	54.3 ± 7.59 (2513)	0.57
Parental smoking	54.2 ± 7.27 (1010)	54.3 ± 7.27 (1492)	0.80
Mother’s drinking	54.4 ± 8.10 (281)	54.3 ± 7.51 (2249)	0.81
Mother’s low education^a^	54.7 ± 7.80 (748)	54.1 ± 7.50 (1779)	0.15
Father’s low education^a^	54.6 ± 7.74 (965)	54.1 ± 7.51 (1554)	0.15

Mean ± standard deviation.

*DBP* diastolic blood pressure, *SBP* systolic blood pressure.

^a^Highest level of education was high school or below.

**Table 4:** Blood pressure between two groups in girls (mmHg).

	Yes (*n*)	No (*n*)	*p* value
SBP, 2 yo			
Past history	90.9 ± 7.81 (90)	90.0 ± 8.09 (2300)	0.30
Gestational hypertension	87.9 ± 9.36 (22)	90.0 ± 8.08 (2423)	0.28
Parental smoking	90.5 ± 8.17 (1042)	59.7 ± 7.98 (1368)	<0.05
Mother’s drinking	90.3 ± 7.99 (253)	90.0 ± 8.10 (2183)	0.60
Mother’s low education^a^	90.3 ± 7.95 (794)	89.8 ± 8.08 (1642)	0.15
Father’s low education^a^	90.5 ± 7.95 (967)	89.6 ± 8.11 (1460)	<0.05
DBP, 2 yo			
Past history	51.8 ± 8.28 (90)	51.6 ± 7.97 (2300)	0.84
Gestational hypertension	50.2 ± 6.58 (22)	51.7 ± 7.99 (2423)	0.44
Parental smoking	51.8 ± 8.07 (1042)	51.6 ± 7.95 (1368)	0.67
Mother’s drinking	51.5 ± 7.90 (253)	51.7 ± 7.98 (2183)	0.78
Mother’s low education^a^	51.4 ± 7.66 (794)	51.7 ± 8.06 (1642)	0.32
Father’s low education^a^	51.7 ± 7.68 (967)	51.6 ± 8.12 (1460)	0.71
SBP, 4 yo			
Past history	92.9 ± 8.90 (90)	93.1 ± 8.06 (2300)	0.89
Gestational hypertension	97.0 ± 9.37 (22)	93.0 ± 8.08 (2423)	<0.05
Parental smoking	93.8 ± 8.21 (1042)	92.5 ± 7.97 (1368)	<0.05
Mother’s drinking	93.2 ± 7.64 (253)	93.1 ± 8.16 (2183)	0.84
Mother’s low education^a^	93.8 ± 8.40 (794)	92.7 ± 7.92 (1642)	<0.05
Father’s low education^a^	93.5 ± 8.25 (967)	92.8 ± 7.99 (1460)	<0.05
DBP, 4 yo			
Past history	53.5 ± 7.12 (90)	54.7 ± 7.88 (2300)	0.20
Gestational hypertension	55.8 ± 4.90 (22)	54.6 ± 7.89 (2423)	0.50
Parental smoking	55.3 ± 7.73 (1042)	54.1 ± 7.93 (1367)	<0.05
Mother’s drinking	54.7 ± 7.37 (253)	54.6 ± 7.92 (2183)	0.93
Mother’s low education^a^	55.2 ± 7.96 (794)	54.4 ± 7.82 (1642)	<0.05
Father’s low education^a^	54.5 ± 7.73 (967)	54.8 ± 8.07 (1460)	0.39

Mean ± standard deviation.

*DBP* diastolic blood pressure, *SBP* systolic blood pressure.

^a^Highest level of education was high school or below.

**Table 5:** Multiple regression analysis.

	*β*	Std. error	*t*	*p* value
*SBP, 2 yo*				
Female	–1.09	0.27	–4.11	<0.05
Birth weight	–1.50 × 10^–4^	3.33 × 10–^4^	–0.45	0.65
Gestational hypertension	–1.10	1.29	–0.86	0.39
Parental smoking	0.88	0.28	3.20	<0.05
Mother’s low education^a^	0.29	0.30	0.96	0.34
Father’s low education^a^	0.21	0.29	0.72	0.47
BMI	0.96	0.12	8.31	<0.05
*DBP, 2 yo*				
Female	0.23	0.26	0.91	0.36
Birth weight	9.00 × 10^–5^	3.25 × 10^–4^	0.28	0.78
Gestational hypertension	–0.85	1.25	–0.68	0.50
Parental smoking	0.37	0.27	1.36	0.17
Mother’s low education^a^	–0.33	0.30	–1.12	0.26
Father’s low education^a^	4.05 × 10^–2^	0.28	0.14	0.89
BMI	0.71	0.11	6.27	<0.05
*SBP, 4 yo*				
Female	–0.56	0.25	–2.25	<0.05
Birth weight	4.74 × 10^–4^	3.09 × 10^–4^	1.53	0.13
Gestational hypertension	3.97	1.30	3.06	<0.05
Parental smoking	0.55	0.26	2.11	<0.05
Mother’s low education^a^	0.82	0.29	2.87	<0.05
Father’s low education^a^	0.49	0.27	1.78	0.075
BMI	0.93	0.11	8.64	<0.05
*DBP, 4 yo*				
Female	0.47	0.24	1.93	0.053
Birth weight	5.46 × 10^–4^	3.04 × 10^–4^	1.80	0.073
Gestational hypertension	0.99	1.29	0.77	0.44
Parental smoking	0.40	0.26	1.58	0.11
Mother’s low education^a^	0.65	0.28	2.32	<0.05
Father’s low education^a^	9.90 × 10^–2^	0.27	0.37	0.71
BMI	0.67	0.11	6.32	<0.05

*BMI* body mass index, *DBP* diastolic blood pressure, *SBP* systolic blood pressure.

^a^Highest level of education was high school or below.

**Fig. 1:**
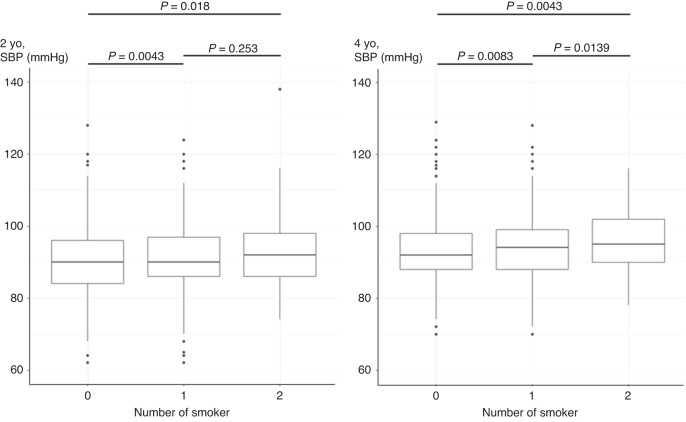
Relationship between number of parental smoking and SBP. SBP demonstrated an increase corresponding to the escalating count of parental smokers.

**Fig. 2:**
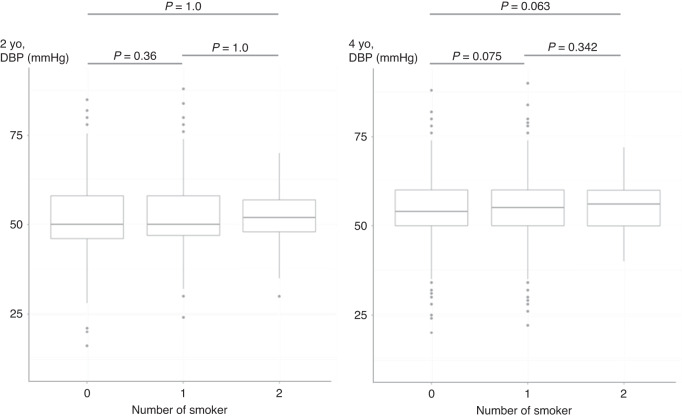
Relationship between number of parental smoking and DBP. DBP demonstrated an increase corresponding to the escalating count of parental smokers.

For the analysis of the three groups of parental smoking status; non-smoker, one smoker, and both smokers, in 2- and 4-year-old SBP and 2-year-old DBP increased as the number of smokers increased, and a significant difference was observed in 2- and 4-year-old SBP (Figs. 1 and 2).

Tables 6 and 7 show the comparison between hypertensive (SBP ≥95th percentile) and non-hypertensive groups. For boys, 95th percentile of SBP was 106 mmHg at both 2 and 4 years old. For girls, 95th percentile of SBP was 104 mmHg at 2 years old and 106 mmHg at 4 years old. The hypertensive group presented significantly higher weight, obesity rate, BSA, and BMI. Among 4-year-old boys, the mother’s lower education was significantly higher in the hypertensive group. Among 4-year-old girls, the mother’s lower education and parental smoking were significantly higher in the hypertensive group.

**Table 6:** Comparison between hypertension and non-hypertension groups in boys.

2 yo	HT (*n* = 113)	Non-HT (*n* = 1861)	*p* value
SBP (mmHg)	109.7 ± 4.46	90.1 ± 7.24	<0.05
Birth weight (g)	3075.9 ± 430.37	3090.0 ± 416.34	0.73
Height (cm)	84.8 ± 3.17	84.5 ± 2.96	0.38
Weight (kg)	12.1 ± 1.18	11.7 ± 1.19	<0.05
Obesity rate	5.40 ± 6.80	2.5 ± 7.32	<0.05
BSA (m^2^)	0.52 ± 0.03	0.5 ± 0.03	<0.05
BMI	16.8 ± 1.08	16.4 ± 1.15	<0.05
TSH (µIU/mL)	2.40 ± 1.11	2.48 ± 1.25	0.53
fT4 (pg/mL)	1.20 ± 0.13	1.22 ± 0.13	0.15
Mother’s height (cm)	158.9 ± 5.42	158.2 ± 5.31	0.17
Mother’s weight (kg)	54.6 ± 9.42	53.1 ± 8.62	0.090
Mother’s BMI	21.6 ± 3.46	21.2 ± 3.23	0.24
Father’s height (cm)	171.2 ± 5.67	171.8 ± 5.77	0.31
Father’s weight (kg)	70.6 ± 11.36	69.0 ± 10.94	0.14
Father’s BMI	24.2 ± 3.90	23.4 ± 3.36	<0.05
Past history (*n*, %)	109 (96.5%)	1766 (94.9%)	0.62
Gestational hypertension (*n*, %)	0 (0%)	26 (1.4%)	0.40
Parent’s smoking (*n*, %)	43 (38.4%)	724 (39.5%)	0.89
Mother’s drinking (*n*, %)	17 (15.0%)	209 (11.3%)	0.29
Mother’s low education^a^ (*n*, %)	36 (31.9%)	525 (28.4%)	0.49
Father’s low education^a^ (*n*, %)	44 (38.9%)	690 (37.4%)	0.81
4 yo	HT (*n* = 141)	Non-HT (*n* = 1977)	*p* value
SBP (mmHg)	109.3 ± 4.04	92.7 ± 6.73	<0.05
Birth weight (g)	3122.5 ± 376.15	3082.9 ± 428.85	0.29
Height (cm)	101.8 ± 4.01	100.2 ± 3.75	<0.05
Weight (kg)	16.7 ± 2.60	15.6 ± 1.69	<0.05
Obesity rate	4.12 ± 10.79	–0.039 ± 6.97	<0.05
BSA (m^2^)	0.68 ± 0.06	0.65 ± 0.04	<0.05
BMI	16.1 ± 1.65	15.5 ± 1.07	<0.05
TSH (µIU/mL)	2.58 ± 1.27	2.48 ± 1.29	0.36
fT4 (pg/mL)	1.23 ± 0.14	1.21 ± 0.13	0.17
Mother’s height (cm)	158.6 ± 5.83	158.2 ± 5.32	0.48
Mother’s weight (kg)	54.7 ± 9.93	53.2 ± 8.66	<0.05
Mother’s BMI	21.7 ± 3.49	21.2 ± 3.25	0.084
Father’s height (cm)	172.0 ± 5.60	171.6 ± 5.79	0.40
Father’s weight (kg)	71.5 ± 12.12	68.9 ± 10.98	<0.05
Father’s BMI	24.1 ± 3.87	23.4 ± 3.42	<0.05
Past history (*n*, %)	131 (92.9%)	1875 (95.6%)	0.21
Gestational hypertension (*n*, %)	3 (2.1%)	17 (0.9%)	0.29
Parental smoking (*n*, %)	51 (37.2%)	763 (39.1%)	0.72
Mother’s drinking (*n*, %)	18 (12.8%)	221 (11.2%)	0.68
Mother’s low education^a^ (*n*, %)	52 (37.1%)	543 (27.6%)	<0.05
Father’s low education^a^ (*n*, %)	58 (41.4%)	717 (36.5%)	0.28

Continuous variables: mean ± standard deviation.

*BMI* body mass index, *BSA* body surface area, *fT4* free thyroxine, *SBP* systolic blood pressure, *TSH* thyroid stimulating hormone.

^a^Highest level of education was high school or below.

**Table 7:** Comparison between hypertension and non-hypertension groups in girls.

2 yo	HT (*n* = 106)	Non-HT (*n* = 1906)	*p* value
SBP (mmHg)	107.5 ± 4.34	89.0 ± 7.08	<0.05
Birth weight (g)	2992.1 ± 397.96	2989.9 ± 398.26	0.96
Height (cm)	83.7 ± 2.70	83.3 ± 2.89	0.11
Weight (kg)	11.7 ± 1.13	11.2 ± 1.15	<0.05
Obesity rate	7.12 ± 8.22	3.2 ± 7.58	<0.05
BSA (m^2^)	0.51 ± 0.03	0.49 ± 0.03	<0.05
BMI	16.7 ± 1.28	16.2 ± 1.17	<0.05
TSH (µIU/mL)	2.29 ± 1.49	2.28 ± 1.19	0.96
fT4 (pg/mL)	1.22 ± 0.14	1.20 ± 0.13	0.069
Mother’s height (cm)	158.4 ± 5.09	158.5 ± 5.52	0.93
Mother’s weight (kg)	54.5 ± 9.59	53.3 ± 8.83	0.21
Mother’s BMI	21.7 ± 3.71	21.2 ± 3.15	0.13
Father’s height (cm)	172.0 ± 5.32	171.9 ± 5.92	0.83
Father’s weight (kg)	71.2 ± 10.58	69.7 ± 11.13	0.17
Father’s BMI	24.1 ± 3.17	23.6 ± 3.41	0.16
Past history (*n*, %)	99 (94.3%)	1832 (96.3%)	0.44
Gestational hypertension (*n*, %)	1 (0.9%)	16 (0.8%)	1
Parental smoking (*n*, %)	52 (50.0%)	798 (42.5%)	0.16
Mother’s drinking (*n*, %)	12 (11.3%)	202 (10.6%)	0.95
Mother’s low education^a^ (*n*, %)	35 (34.0%)	591 (31.1%)	0.44
Father’s low education^a^ (*n*, %)	48 (47.1%)	732 (38.7%)	0.11
4 yo	HT (*n* = 152)	Non-HT (*n* = 1944)	*p* value
SBP (mmHg)	109.5 ± 4.39	91.8 ± 6.81	<0.05
Birth weight (g)	3002.4 ± 431.01	2992.9 ± 393.42	0.78
Height (cm)	99.8 ± 3.90	99.3 ± 3.69	0.14
Weight (kg)	16.0 ± 2.15	15.3 ± 1.72	<0.05
Obesity rate	4.10 ± 9.86	0.83 ± 7.37	<0.05
BSA (m^2^)	0.66 ± 0.05	0.64 ± 0.04	<0.05
BMI	16.0 ± 1.52	15.5 ± 1.14	<0.05
TSH (µIU/mL)	2.37 ± 1.30	2.27 ± 1.23	0.35
fT4 (pg/mL)	1.20 ± 0.14	1.20 ± 0.13	0.75
Mother’s height (cm)	158.8 ± 5.46	158.4 ± 5.52	0.44
Mother’s weight (kg)	54.1 ± 8.95	53.3 ± 8.83	0.32
Mother’s BMI	21.4 ± 3.30	21.2 ± 3.21	0.45
Father’s height (cm)	170.9 ± 6.23	171.9 ± 5.82	0.057
Father’s weight (kg)	71.1 ± 11.21	69.7 ± 11.13	0.12
Father’s BMI	24.3 ± 3.40	23.6 ± 3.40	<0.05
Past history (*n*, %)	141 (94.6%)	1857 (96.3%)	0.42
Gestational hypertension (*n*, %)	3 (2.0%)	16 (0.8%)	0.32
Parental smoking (*n*, %)	79 (52.7%)	786 (41.0%)	<0.05
Mother’s drinking (*n*, %)	11 (7.2%)	208 (10.7%)	0.22
Mother’s low education^a^ (*n*, %)	61 (40.4%)	596 (30.8%)	<0.05
Father’s low education^a^ (*n*, %)	65 (42.8%)	758 (39.3%)	0.46

Continuous variables: mean ± standard deviation.

*BMI* body mass index, *BSA* body surface area, *fT4* free thyroxine, *SBP* systolic blood pressure, *TSH* thyroid stimulating hormone.

^a^Highest level of education was high school or below.

## Discussion

In the present study, we showed risk factors of hypertension in toddlers using the data obtained from the nationwide large birth cohort study in Japan. The BP range in this study was broadly similar to that in the report from the United States.^4^ In addition, compared with the recent reports of school-age children in Japan,^5–7^ the BP range in this study was lower than the BP indicated by them. The result was consistent with the knowledge that BP in children generally increases with age^5^ as we have also shown in this study that the range of BP in 4 years old was higher than that in 2 years old.

We presented that obesity, parental smoking, and lower education were associated with toddler’s hypertension. Simonetti et al. also reported that these factors were at risk for hypertension in a study of preschool children aged 5–6 years.^21^ Other studies also reported that childhood obesity was a risk factor for pediatric hypertension.^16–20^ Sorof et al. reported that obese children had more systolic hypertension in children aged 12–16 years (33% vs 11%, *p* < 0.001).^18^ In another study, BMI was shown to be strongly associated with systolic hypertension in children aged 10–19 years, and that SBP increased incrementally with each increase in BMI (*p* < 0.001).^20^ Previous reports showed the association between obesity and hyperactivity in the sympathetic nervous system with increased heart rate, BP,^18,36^ and loss of vascular function.^37^ Furthermore, a previous report showed that adipose tissue promoted renin-angiotensin system, which led to hypertension in obesity.^38^ We also speculated that obese children tended to have unhealthy lifestyles, which might cause an increase in BP. Since Erdal et al. reported that the relationship between obesity and hypertension in children varied according to sex,^39^ we considered the possibility that the effects of obesity or environmental factors on BP might vary between boys and girls. In fact, there were differences in SBPs between boys and girls in this study (Table 1). Therefore, we conducted the analysis by separating them according to sex.

Previous studies reported that smoking was a risk factor for hypertension in adults.^14,15,40–43^ These suggested that smoking caused excessive activity of the sympathetic nervous system, oxidative stress, vasoconstriction, and long-term arteriosclerosis, which lead to hypertension.^40,41^ Some studies reported that second-hand smoking was also a risk factor for hypertension.^30–34^ Li et al. reported that second-hand smoking raised the risk of hypertension (adjusted odds ratio: 1.99, 95% CI 1.16–3.39) which increased incrementally with the frequency of second-hand smoking compared to nonsmoking women.^31^ Yang et al. showed an association between husbands’ smoking and the prevalence of hypertension in women.^33^ Few reports showed the relationship between second-hand smoking and children’s BP.^44,45^ Zhang et al. showed that girls who were exposed to parental smoking were more likely to have hypertension in a study of children aged seven to 18 years.^45^ Environmental tobacco smoke exposure increased air chemicals such as nicotine, myosmine, solanesol and 3-ethenylpyridine,^46–49^ and second-hand smoking was associated with urine cotinine level.^46,47,49^ Therefore, we considered that second-hand smoking would increase BP with similar mechanisms in active smoking as early as 2 years old shown in our study. A previous report also showed that maternal second-hand smoking influenced GH.^50^ Other reports showed that GH was a risk factor for hypertension in adolescents,^51,52^ and our study also showed that GH affected hypertension in 4-year-olds. Some animal studies showed that prenatal stress might cause dysregulation of both maternal and fetal glucocorticoids and affect the actions of angiotensin 2 and noradrenaline.^53–55^

It has been reported that parents with lower education tended to have “unhealthy” eating habits,^56^ such as high-salt with less vegetables and fruits diet.^11,57–59^ Eckel et al. reported exercise therapy reduced SBP by 2–5 mmHg and DBP by 1–4 mmHg.^60^ Several studies showed that sleep disorders and psychosocial stress increased the incidence of hypertension.^12,61–63^ Therefore, we speculated that lower health literacy might interfere with exercise habits and good sleep quality, which resulted in increased BP.

In multiple regression analysis, the mother’s low education level was associated with significant differences in 4-year-old BP, while there was no significant difference at 2 years of age. On the other hand, a significant difference in parental smoking was observed even at the age of 2 years. We speculated that the “unhealthy lifestyles” affect BP after some years whereas second-hand smoking had a particularly strong and immediate effect on BP.

This study had some limitations. First, several items of dataset were based on the questionnaires filled out by parents. Second, it is difficult to accurately measure the BP of children. To obtain the accurate BP value as possible, we repeated BP measurement three times according to the JSH 2014 and previous reports.^64,65^ However, some children were unable to participate in the three measurements or were unable to measure multiple times. In addition, because we excluded children whose BP could not be measured accurately even once, there might be a selection bias for children with hyperactivity or irritability. Third, exposure to environmental factors obtained from questionnaires, such as cigarette smoke, at the time of BP measurement may differ from the time of questioning. Finally, because this study was a questionnaire survey targeting the general population, there might be a selection bias for parents with higher health literacy at the time of participating in the study.

## Conclusion

We showed the risk factors of hypertension at 2 and 4 years old in Japan. Obesity, parental smoking and lower education were associated with toddler’s hypertension. Especially, second-hand smoking had a significant effect on BP even in early toddlers. We emphasize the importance of avoiding second-hand smoking from early infancy to prevent future lifestyle-related illnesses including hypertension.

### Supplementary information

Supplementary table 1

## Data Availability

The data that support the findings of this study are available from The Japan Environment and Children’s Study Group but restrictions apply to the availability of these data, which were used under license for the current study, and so are not publicly available. Data are however available from the authors upon reasonable request and with permission of The Japan Environment and Children’s Study Group.
