# Supplementary material for: Environments affect blood pressure in toddlers: The Japan Environment and Children’s Study
Source: Pediatr Res. 2023 Aug 26;95(1):367–76. doi: 10.1038/s41390-023-02796-8 (PMC10798899; doi:10.1038/s41390-023-02796-8)
Supplement: Supplementary file 1 — Supplementary table 1 [file 41390_2023_2796_MOESM1_ESM.pdf]

Supplementary table 1. Blood pressure levels by condition

	Resting/sleeping	Crying/moving	P value
2 yo, boys SBP (mmHg)	90.6 ± 8.28	95.6 ± 11.3	< 0.05
2 yo, boys DBP (mmHg)	51.6 ± 8.01	53.9 ± 10.0	< 0.05
4 yo, boys SBP (mmHg)	93.4 ± 7.95	95.8 ± 8.87	< 0.05
4 yo, boys DBP (mmHg)	54. 5 ± 7.73	53.5 ± 9.35	< 0.05
2 yo, girls SBP (mmHg)	90.0 ± 8.09	95.1 ± 11.8	< 0.05
2 yo, girls DBP (mmHg)	51.7 ± 7.97	53.7 ± 10.0	< 0.05
4 yo, girls SBP (mmHg)	93.1 ± 8.10	96.1 ± 9.40	< 0.05
4 yo, girls DBP (mmHg)	54.6 ± 7.86	53.1 ± 9.11	< 0.05

mean ± standard deviation. DBP, diastolic blood pressure; SBP, systolic blood pressure.
